# Supplementary material for: Standard machine learning approaches outperform deep representation learning on phenotype prediction from transcriptomics data
Source: BMC Bioinformatics. 2020 Mar 20;21:119. doi: 10.1186/s12859-020-3427-8 (PMC7085143; doi:10.1186/s12859-020-3427-8)
Supplement: Supplementary file 1 — Additional file 1 Supplementary Figures and Tables. [file 12859_2020_3427_MOESM1_ESM.pdf]

Supplementary Figures and Tables

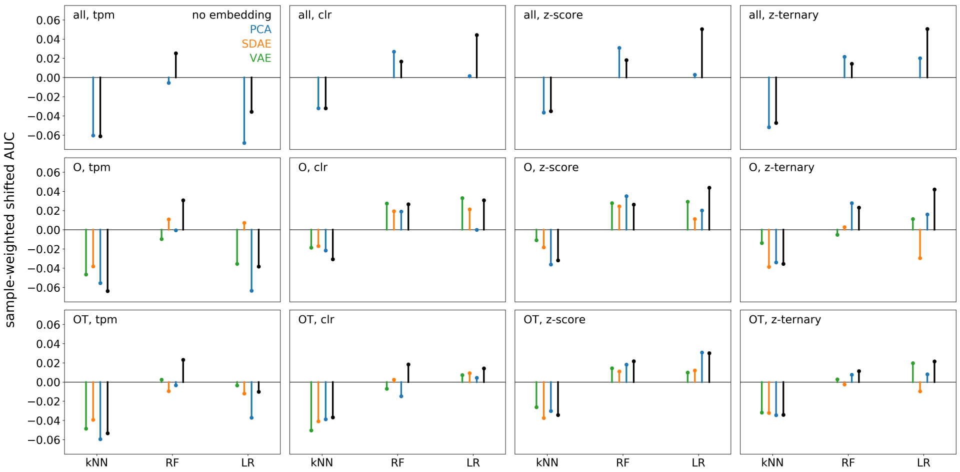

Figure 1 Performance over all model types. The binary task performance of each unique model type is shown for each combination of gene set, transform, supervised model, and unsupervised model. Each plot is a specific gene set and transform combination, and inside each plot results are grouped by supervised model and colored by unsupervised model. The performance shown is the average of shifted AUCs across binary tasks, weighted by the number of samples in each task to reduce the effect of fluctuations in tasks with fewer samples. The best results come from using all genes without an unsupervised embedding.

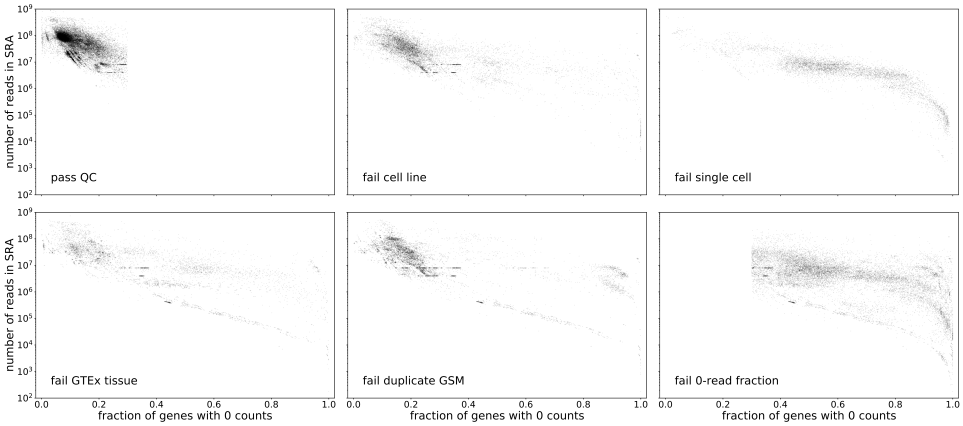

Figure 2 Effect of the quality control cuts. Gene expression samples plotted in terms of two quality metrics, the number of reads and the fraction of genes with zero reads. The upper left plot shows each sample in the recount2 database passing quality cuts. The remaining plots show samples failing each of the quality cuts. The cuts remove a swath of samples whose characteristics are distinct from the bulk of high-quality samples retained in the dataset.

Author details

References

1. Kingma, D.P., Ba, J.: Adam: A Method for Stochastic Optimization. arXiv:1412.6980 [cs] (2014). 1412.6980
2. Bowman, S.R., Vilnis, L., Vinyals, O., Dai, A.M., Jozefowicz, R., Bengio, S.: Generating Sentences from a Continuous Space. arXiv:1511.06349 [cs] (2015). 1511.06349
3. Glorot, X., Bengio, Y.: Understanding the difficulty of training deep feedforward neural networks. Proceedings of the thirteenth international conference on artificial intelligence and statistics, 249–256 (2010)
4. Ioffe, S., Szegedy, C.: Batch Normalization: Accelerating Deep Network Training by Reducing Internal Covariate Shift. arXiv preprint arXiv:1502.03167 (2015)

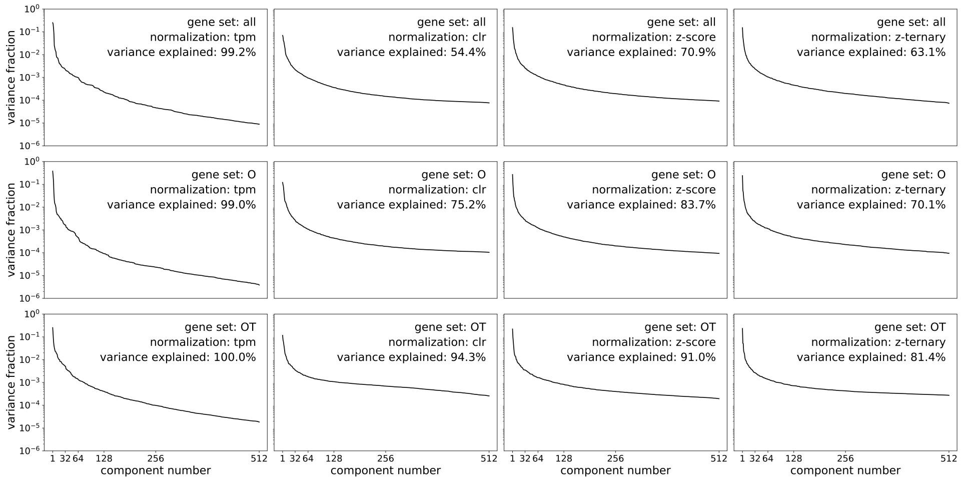

Figure 3 Variance explained by PCA. Per-component variance explained by PCA for each gene set and normalization combination. The total variance explained by all 512 components is displayed on each plot. The majority of variance is captured by the PCA in all cases; in some nearly all variance is captured.

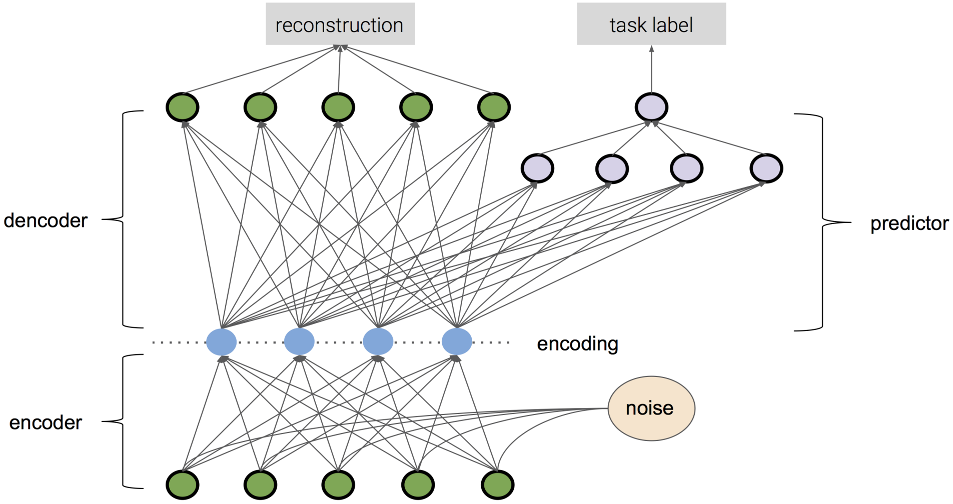

Figure 4 Semi-supervised model schematic. The semi-supervised model consists of a denoising autoencoder coupled to one or more predictors. The training loss is a combination of reconstruction error and classification error.

gene set	layer dimensions
O	17970 - 2048 - 1024 - 512 - 1024 - 2048 - 17970
OT	1530 - 1530 - 1024 - 512 - 1024 - 1530 - 1530

init. weight std. dev.	noise std. dev.	epochs	batch size	init. LR	LR step	LR gamma	l_2-coeff.
0.01	0.3	500	50	0.0001	50	0.8	0

Table 1 SDAE Architectures and Hyperparameters.

- All data was standardized before training.
- Weights were intialized randomly according to a central Gaussian distribution of standard deviation `init. weight std. dev.`.
- The learning rate was reduced from `init. LR` by a factor of `LR gamma` every `LR step` epochs.
- All activations were ReLU except for the final layer, which was *linear* or *hardtanh* in the case of Z-ternary normalization.
- We saw no perceived benefit from l_2-regularization over-against selection of the noise level, and so simply fixed the l_2 coefficient l_2`-coeff.` to 0. The value of `noise std. dev.` was selected by assessing validation performance over a range of values from 0 to 0.5.
- All SGD used ADAM [1] with parameters $(0.5, 0.999)$.
- Models were first trained in a greedy-layerwise fashion before being trained end-to-end. Both training eras used the same set of hyperparameters.

gene set	layer dimensions
O	17970 - 1024 - 1024 - 1024 - 1024 - 1024 - 17970
OT	1530 - 1024 - 1024 - 1024 - 1024 - 1024 - 1530

epochs	batch size	learning rate	KL-annealing rate
10000: tpm<br>1000: clr, Z-score, Z-ternary	100	0.0001	0.01 per epoch

Table 2 VAE Architectures and Hyperparameters.

- Weights were intialized with a centered Gaussian distribution with a standard deviation equal to the inverse of the number of input features.
- Models were trained with the KL-annealing rate [2] moving from 0 to 1 linearly over the first 100 epochs.
- The learning rate was held constant in training.
- No additional regularization was performed due to a strong correlation between training and validation loss.

epochs	batch size	init. LR	LR step	LR gamma
200: binary<br>300: multiclass	floor(num. samples / 10)	0.001	10	0.9

Table 3 Logistic Regression Hyperparameters.

- Weights were initialized randomly according to the standard (Xavier) Glorot normal [3] prescription.
- The learning rate was reduced from `init. LR` by a factor of `LR gamma` every `LR step` epochs.
- The l_2`-coeff` value is selected by cross-validation over the range 10^{-6} to 10^{3} in logarithmic steps of 10.
- All SGD used ADAM [1] with parameters (0.5, 0.999).

epochs	batch size	init. LR
500	floor(num. samples / 5)	0.00001

Table 4 Cox Proportional Hazards Hyperparameters.

- The neural network model consists of a batch-normalization layer [4] followed by a single, linear fully-connected to compute the relative risk function.
- Weights were initialized randomly according to the standard (Xavier) Glorot normal [3] prescription.
- The learning rate was held constant during training.
- The l_2`-coeff` value is selected by cross-validation over the range 10^{-6} to 10^{3} in logarithmic steps of 10.
- All SGD used ADAM [1] with parameters (0.5, 0.9).

gene set	autoencoder layer dimensions	predictor layer dimensions
O	17970 - 512 - 17970	512 - [num labels]
OT	1530 - 512 - 1530	512 - [num labels]

epochs	unlabeled batch size	labeled batch size	noise std. dev.	AE l_2-coeff.
200	50	10	0.3	[0, 0.0001, 0.01, 0.1]

predictor l_2-coeff.	init. LR	LR step	LR gamma	predictor strength
[0, 0.001, 0.1]	0.0001	10	0.8	[0, 0.1, 1]

Table 5 Semi-supervised Model Architectures and Hyperparameters.

- **All data was standardized before training.**
- **Brackets indicate different possible values. All possible combinations of parameters were tried with the best parameter set chosen by virtue of performance on held-out half of the divided predictive tasks.**
- **Weights were initialized randomly according to a central Gaussian distribution with standard deviation of 0.01.**
- **The learning rate was reduced from `init. LR` by a factor of `LR gamma` every `LR step` epochs.**
- **All SGD used ADAM [1] with parameters (0.9, 0.9).**
